# Beyond the Harmonic Oscillator; Highlights of Selected Studies of Vibrational Potential Energy Functions

**DOI:** 10.3390/molecules30071492

**Published:** 2025-03-27

**Authors:** Esther J. Ocola, Jaan Laane

**Affiliations:** 1 Department of Chemistry, Texas A&M University, College Station, TX 77843-3255, USA; 2 Institute for Quantum Science and Engineering, Texas A&M University, College Station, TX 77843-4242, USA

**Keywords:** potential energy functions, molecular inversion, ring puckering, anomeric effect, pseudorotation, internal rotation, π-type hydrogen bonding

## Abstract

Although the harmonic oscillator model has found wide use in physics and chemistry, there are more interesting potential energy functions (PEFs) which can tell us a great deal about molecular structure and energetics. In the present work, we show that for selected systems simple one- and two-dimensional potential functions can be used to very accurately fit detailed spectroscopic data and provide extensive additional information. Results for molecular inversion, ring puckering, the anomeric effect, pseudorotation, triplet-state puckering, internal rotation, and π-type hydrogen bonding in ground and excited electronic states are presented.

## 1. Introduction

The harmonic oscillator (HO) potential energy function V=12kx2 has found wide use in physics and chemistry, and, in particular, in molecular spectroscopy. This is in part due to the fact that the Schrödinger Equation can be solved exactly for the energy levels of the HO. However, there are other forms of potential energy functions (PEFs) and potential energy surfaces that are needed to describe particular molecular systems, and many of these are arguably more interesting. Laane et al. have been investigating these for six decades and periodically published reviews of the work [1,2,3,4,5,6,7,8]. Among these are double-minimum potential functions for cyclic ring inversions dominated by quartic terms. Another type of one-dimensional potential function is that for analyzing the internal rotations of molecules. These have periodic potential energy functions. In the present paper, we present a selection of experimental and theoretical results of work previously published and mostly from our own laboratory.

## 2. Experimental and Theoretical Results

### 2.1. Harmonic Oscillator

**Figure 1:**
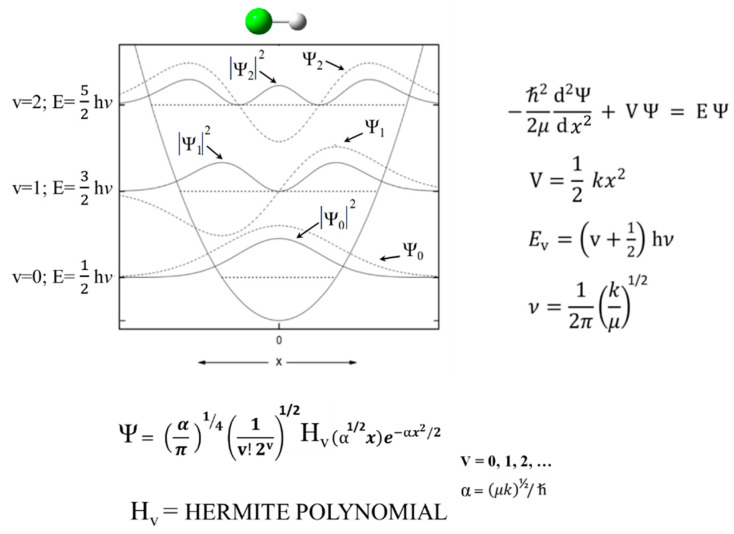
Harmonic oscillator potential energy function and solutions.

**Figure 2:**
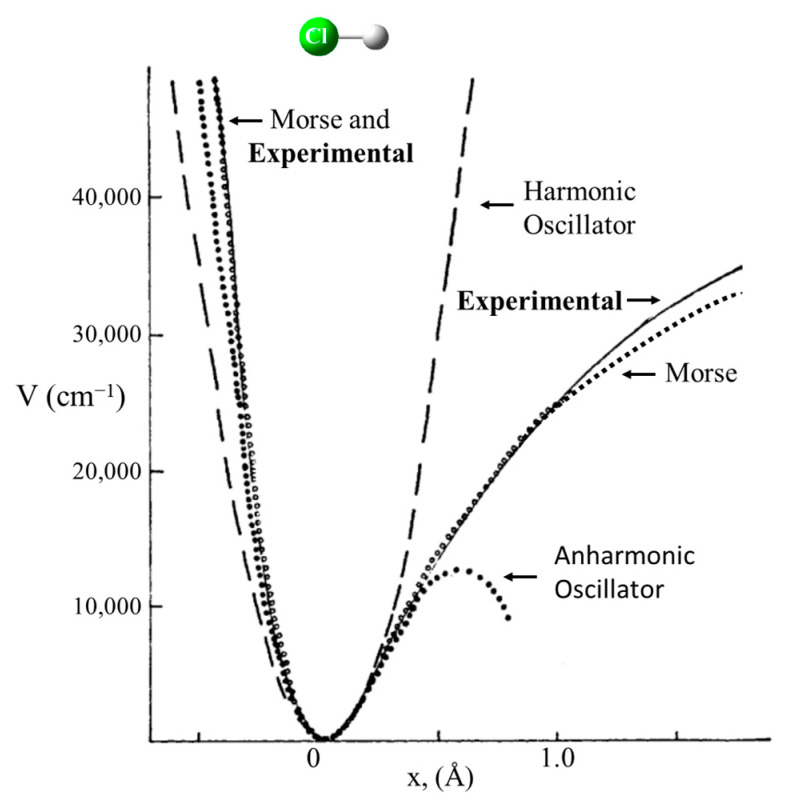
Harmonic oscillator corrected for anharmonicity.

Schrödinger himself recognized that his equation could be solved exactly for the harmonic oscillator [9]. Figure 1 shows the Schrödinger Equation along with the solutions and a diagram of the HO potential function along with the energy levels and wave functions. This has been widely used to calculate force constants for the vibrations of diatomic molecules and for bond stretching vibrations of larger molecules when their frequencies are sufficiently higher than the other vibrations. However, it was recognized early on that, unlike for the harmonic oscillator, the energy spacing for the upper levels of real molecules progressively decreased as the dissociation limit was approached. This anharmonicity arises from the fact that the potential energy, V, does not approach infinity as the bond distance gets very large. Figure 2 compares the harmonic oscillator function to the Morse Potential, which was developed to more accurately calculate the energy levels for the upper states.

### 2.2. Ammonia Inversion

**Figure 3:**
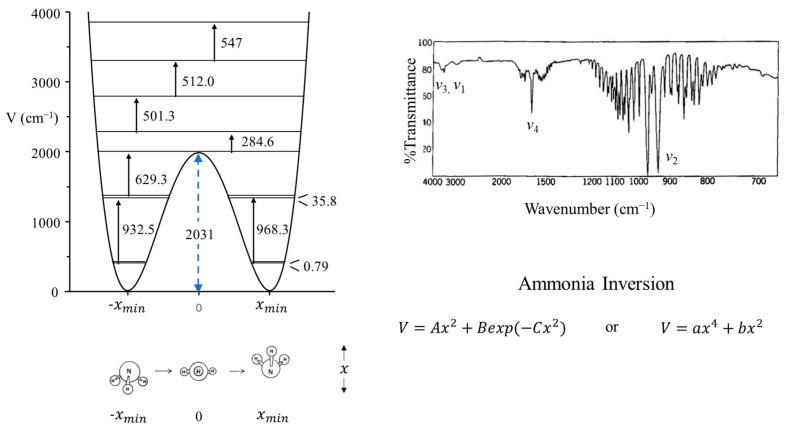
Vapor-phase infrared spectrum of ammonia and its vibrational potential energy function and energy levels for the inversion. The inversion doubling of *v*_2_ can be seen.

The inversion vibration of ammonia can be studied as a one-dimensional problem since it has A_1_ symmetry and has a frequency very much lower than the symmetric N-H_3_ stretching vibration of the same symmetry. The potential function for the inversion has a double minimum for the two equivalent inverted structures and a barrier for the planar configuration. This results in inversion doubling. Figure 3 shows the infrared spectrum of gaseous ammonia, and the doubling can be seen for the inversion, *v*_2_. Figure 3 also shows the observed energy spacings and the calculated potential function which best fits the experimental data [10]. This shows the barrier to planarity to be 2031 cm^−1^ or 5.8 kcal/mole. It also demonstrates how such potential energy functions are invaluable for providing energy and structural information on molecules. Coon and co-workers [11] used the following function to fit the data.

(1) $$V=Ax^{2}+Bexp(-Cx^{2})$$

where x is the inversion coordinate and A, B, and C are adjusted potential energy constants. Laane [12] showed that the following potential energy function could also do a reasonably good job of fitting the experimental data.

(2) $$V=ax^{4}+bx^{2}$$

where the constant b is negative.

### 2.3. Carbonyl Inversion in the Excited State

**Figure 4:**
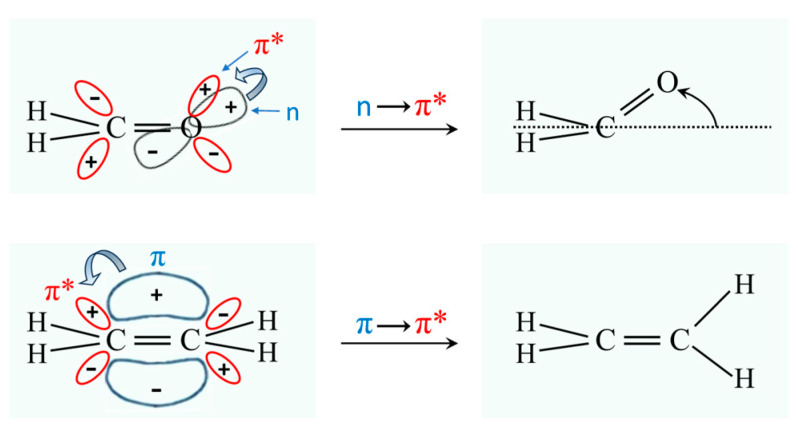
Structural changes for formaldehyde and ethylene following electronic transitions. The * means it is an antibonding orbital.

**Figure 5:**
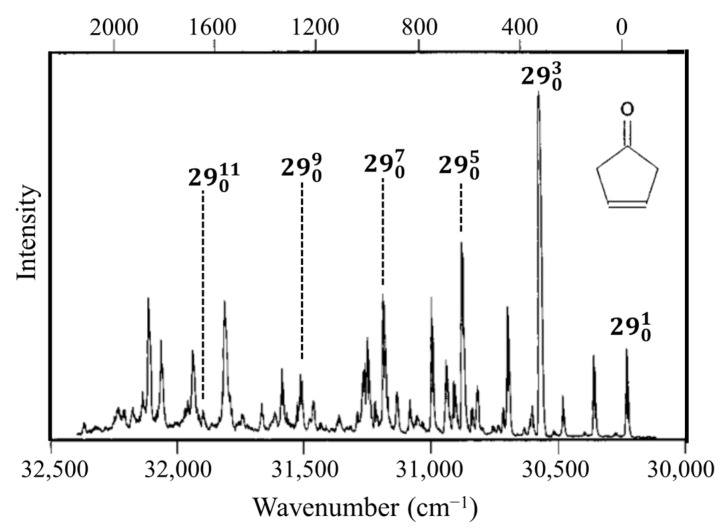
Fluorescence excitation spectrum of 3-cyclopentene-1one. *v*_29_ is the carbonyl inversion vibration.

**Figure 6:**
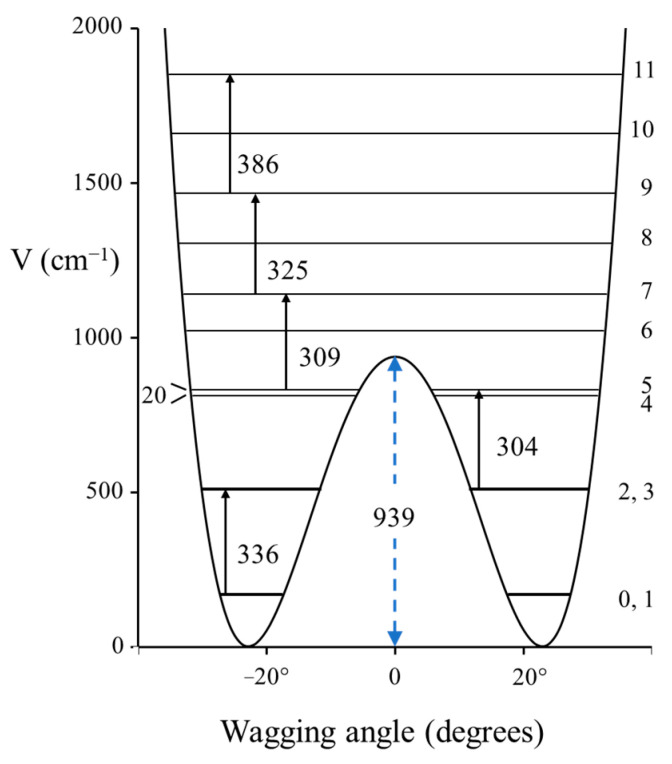
Carbonyl inversion potential energy function for 3-cyclopentene-1one.

Molecules typically change structure after an electronic transition takes place, since electron distributions are changed. Figure 4 shows how the structures of formaldehyde and ethylene change upon such transitions. For formaldehyde, the lowest energy structure for the S_1_(n,π*) excited state has the carbonyl oxygen out of the plane of the other three atoms. Hence, the carbonyl inversion (or carbonyl out-of-plane wagging) vibration will have a double-minimum PEF with a barrier at the planar conformation. Similarly, carbonyl groups in larger molecules will also have inversions governed by double-minimum PEFs. The Laane laboratory has investigated the fluorescence excitation spectra of a number of ring molecules containing carbonyl groups and determined their potential energy functions for the S_1_(n,π*) states. Figure 5 shows the spectrum of 3-cyclopenten-1-one, and Figure 6 shows the carbonyl inversion PEF derived from that [13]. Table 1 presents the barriers to inversion in the excited state for this molecule as well as for several other cyclic molecules [14]. The barrier for 2-cyclopenten-1-one does not exist, since the conjugation is still present between the C=C and C=O groups to some extent in the S_1_(n,π*) state. For the other molecules, the barrier increases with angle strain within the ring. As can be seen, the determinations of the PEFs again provide both structural and relative energy data for these molecules.

### 2.4. Ring Puckering

**Figure 7:**
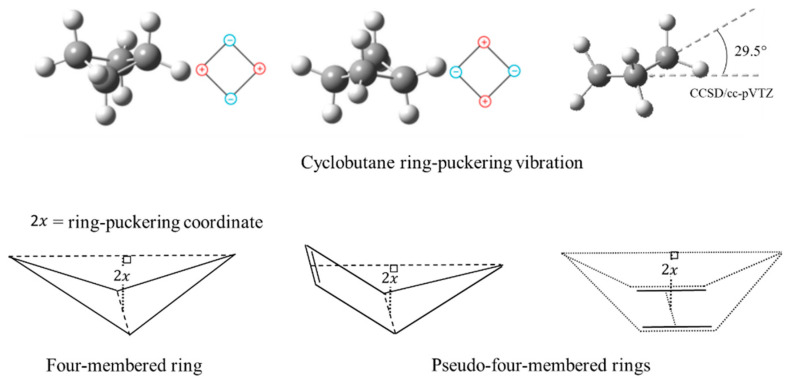
Ring-puckering vibrations for four-membered rings and pseudo-four-membered rings.

In 1945, R. P. Bell [15] postulated that the ring-puckering vibration of cyclobutane should be governed by a quartic potential energy function:

(3) $$V=ax^{4}$$

where x is the ring-puckering coordinate reflecting the out-of-plane displacement. Bell did not present a mathematical explanation for this. Laane [16] later demonstrated that the function should have the form of Equation (2) if the angle-bending force constants are assumed to have quadratic dependence. He showed that angle strain resulted in the $x^{4}$ quartic term but also made a smaller positive contribution to the $x^{2}$ term. Negative contributions to the quadratic term generally arise for torsional forces such as CH_2_-CH_2_ interactions. Laane and Lord [17] also postulated that the ring puckering of so-called “pseudo-four-membered-rings”, such as cyclopentene and 1,4-cyclohexadiene, could be investigated by one-dimensional PEFs. Figure 7 depicts these ring-puckering vibrations.

Bell’s prediction of quartic oscillator functions excited the spectroscopic community in the 1960s when far-infrared equipment started to become available. References to the early work can be found in our previous reviews [1,2,3,4,5,6,7,8]. A dilemma at the time was that large-frame computers were slow and had memory capacities of only 512 K. Moreover, the wave equation for the potential function in Equation (2) could not be solved exactly and needed approximation methods. To facilitate the fitting of ring-puckering spectra, Laane [12] generated a table of energy levels for the reduced potential energy function:

(4) $$V=A(Z^{4}+BZ^{2})$$

where the constants A and B can be related to a and b in Equation (2) if the reduced mass for the wave equation is known. Similarly, Z could be related to the puckering coordinate, x. At that time, the problem was that there were no methods for calculating the reduced mass, which, as it turns out, is also coordinate-dependent. Harthcock and Laane [18,19] later did develop methods for reduced mass calculations, but in the 1960s and 1970s these were not available.

**Figure 8:**
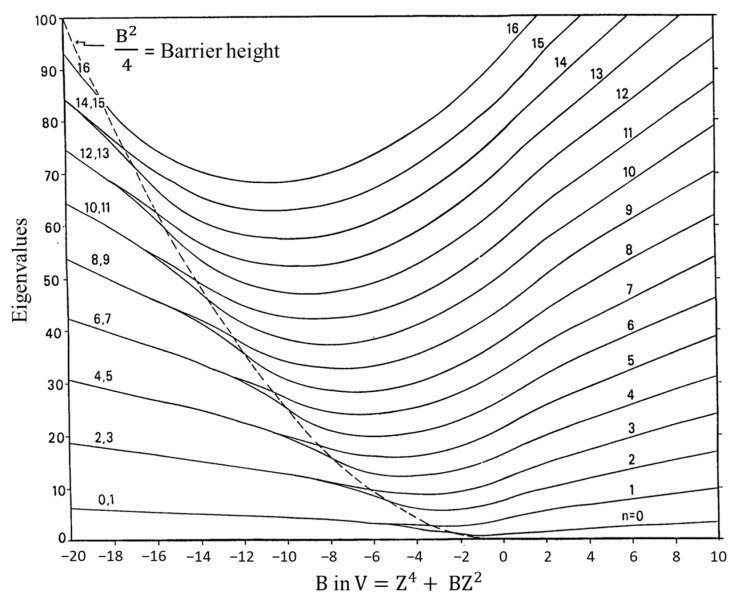
Energy level for the reduced potential energy function V = Z^4^ + BZ^2^ as a function of B.

**Figure 9:**
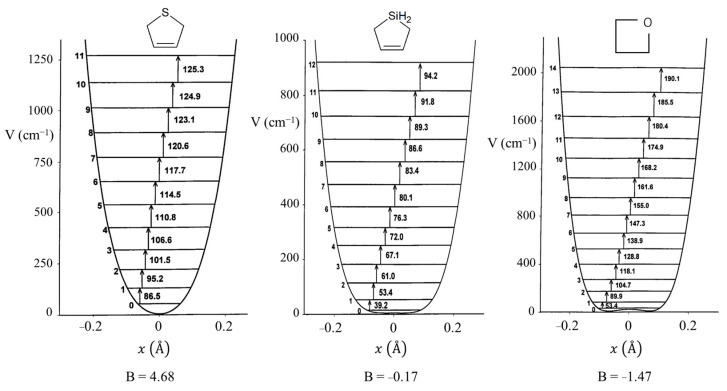
Ring-puckering potential energy functions for three nearly planar molecules.

**Figure 10:**
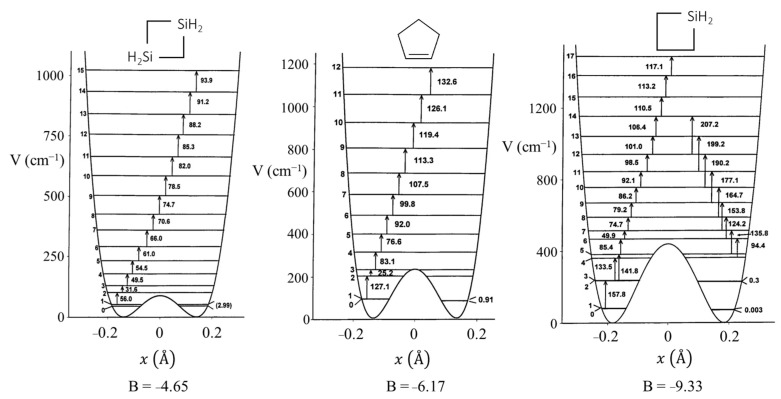
Ring-puckering potential energy functions for three puckered molecules.

**Figure 11:**
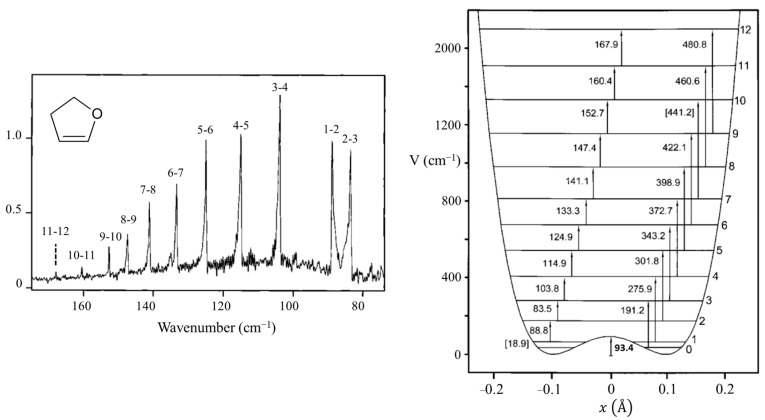
Far-infrared spectrum, potential energy function, and transitions for 2,3-dihydrofuran.

Figure 8 presents a graph of how the energies of the quantum states vary as a function of the constant B in Equation (4). Once B is determined from relative energy spacings, the constant A can be used as a scaling factor to fit the spectrum. As can be seen, as the barrier B^2^/4 increases, the energy levels begin to double up. Equation (4) was used to fit the far-infrared spectra of many molecules [12], and Figure 9 shows the potential functions and transitions for three nearly planar molecules for which the angle strain and torsional forces affecting the b coefficient in Equation (2) nearly balance out. Figure 10 shows the functions and transitions for molecules with greater torsional interactions giving rise to sizable barriers. The fitting of the observed spectroscopic data with Equation (2) is remarkably good, especially after the coordinate-dependent reduced mass is correctly calculated. To demonstrate this, Figure 11 presents the far-infrared spectrum of 2,3-dihydrofuran along with its puckering potential function and transitions [20]. The small barrier to planarity arises from the single CH_2_-CH_2_ interaction. Table 2 shows that the two-parameter potential function fits the observed data with a better than 1% accuracy.

Since about the year 2000, ab initio calculations have progressively improved so that they can fairly accurately predict potential energy functions for the ring-puckering motions. Ocola and Laane [21], in 2020, presented a comparison of ab initio CCSD/cc-pVTZ and MP2/cc-pVTZ results with experimental ones. As can be seen in Table 3, in most cases the CCSD/cc-pVTZ calculation gives better agreement with the experimental data.

### 2.5. Anomeric Effect

**Figure 12:**
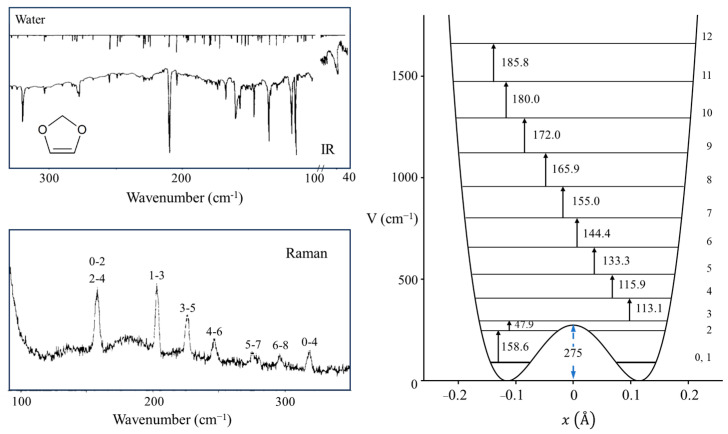
Far-infrared and Raman spectra and potential energy function of 1,3-dioxole.

**Figure 13:**
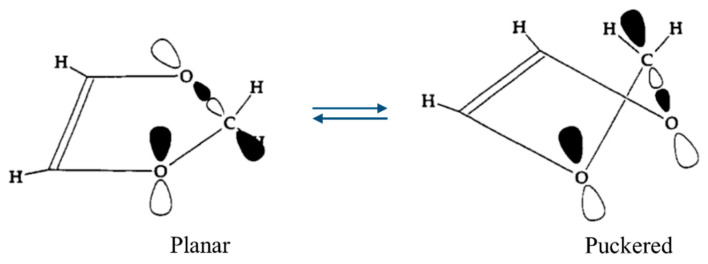
Orientation of the n and σ* orbitals involved in the anomeric effect for planar and puckered conformations of 1,3-dioxole.

One of our ring-puckering investigations proved perhaps to be the best demonstration of the anomeric effect which occurs for molecules containing a XCH_2_Y grouping, where X or Y = O, S, or Se. Figure 12 presents the infrared and Raman spectra of 1,3-dioxole [22], and Figure 13 shows the ring-puckering PEF determined from the data. The potential energy function demonstrates that the energy minima correspond to puckered structures brought on by the anomeric effect. Figure 13 shows how the puckering is brought about by the overlap of the non-bonded oxygen n orbital with the σ* orbital of the other O-C linkage.

**Figure 14:**
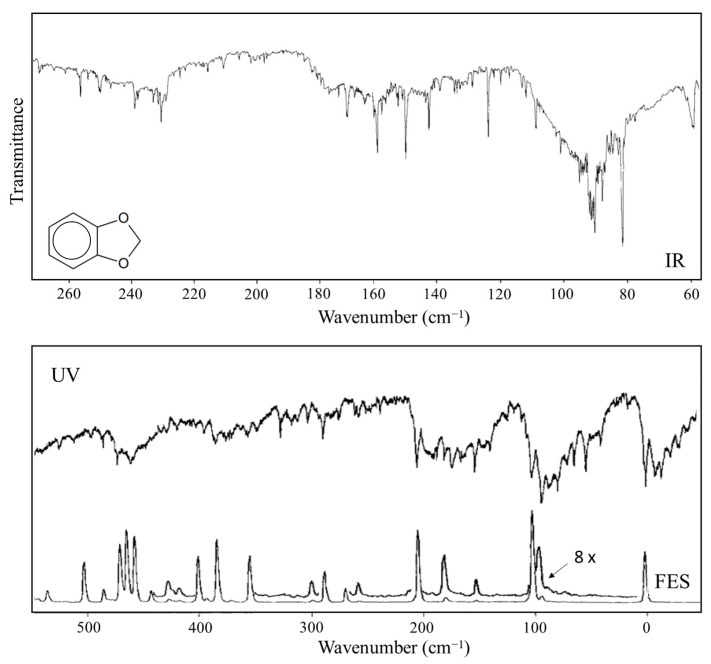
Far-infrared and excited-state spectra of 1,3-benzodioxole. The excited-state spectrum band origin is at 34,789.8 cm^−1^.

**Figure 15:**
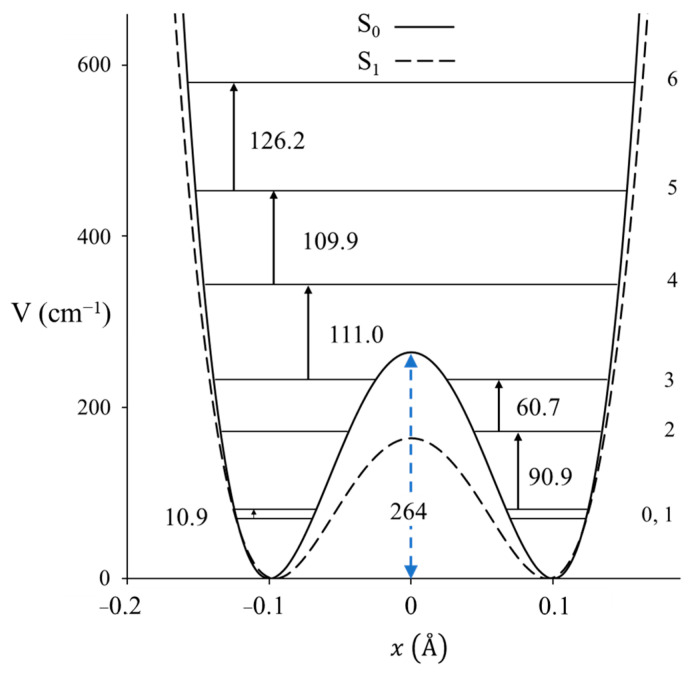
One-dimensional ring-puckering potential energy functions of 1,3-benzodioxole in its ground and excited S_1_(π,π*) states.

Laane et al. have also reported the infrared and excited-state spectra for 1,3-benzodioxole [23,24] and showed that the anomeric effect again is responsible for the non-planarity of the five-membered ring. The data are actually best fit with a two-dimensional potential energy surface in terms of the ring-puckering and the ring-flapping vibrations. Figure 14 shows the spectra, and Figure 15 shows the one-dimensional potential energy along the puckering coordinate calculated for the two electronic states. The lower barrier to planarity for the excited state is attributed to competition for the oxygen non-bonded n orbital and the benzene ring π system.

The experimental results for 1,3-dioxole along with ab initio CCSD/cc-pVTZ and MP2/cc-pVTZ calculations allowed us to calculate the magnitude of the anomeric effect for different XCH_2_Y arrangements, and the results are shown in Table 4 [25]. We believe that these results provide a greatly improved understanding of the anomeric effect for the different atoms in the XCH_2_Y linkages.

### 2.6. Pseudorotation

**Figure 16:**
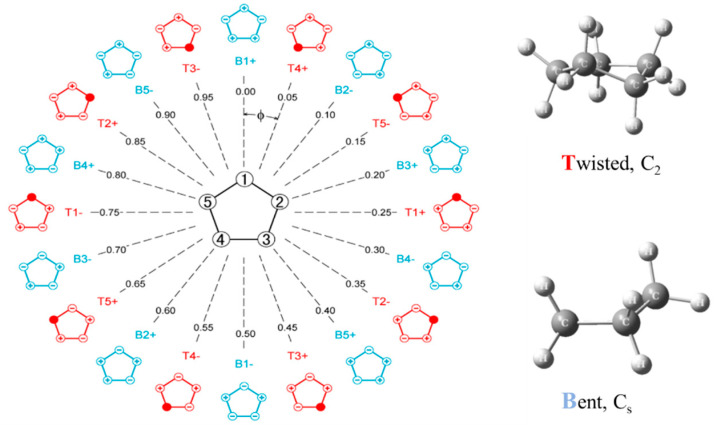
Pseudorotation of cyclopentane.

**Figure 17:**
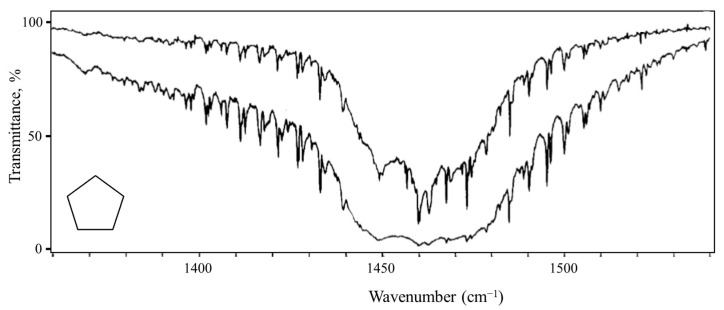
Pseudorotation combination band spectrum of cyclopentane.

Pitzer and co-workers [26,27,28], as early as 1947, postulated that cyclopentane should undergo free pseudorotation. The basic concept is that the out-of-plane bending and twisting vibrations, which would be degenerate for a planar cyclopentane molecule, can be transformed to a radial mode and a pseudorotational mode which “pseudorotates” from a bent structure to a twisted structure to another bent structure, and so on. Figure 16 shows this progression. The theory also predicted that the pseudorotation would be nearly free, with little or no energy differences between the bent and twisted conformations, and this would result in energy levels similar to pure rotation. Experimental evidence for this was first confirmed by Durig and Wertz [29], who reported low-resolution infrared combination band spectra in the CH_2_ deformation region. High-resolution spectra were later published by Bauman and Laane [30], who also reported radial bands and the combination bands for several isotopic species. Figure 17 shows the higher-resolution spectrum.

**Figure 18:**
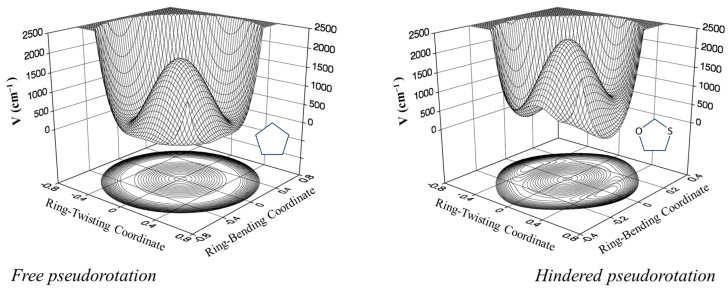
Two-dimensional potential energy surfaces for cyclopentane and 1,3-oxathiolane.

Since what we have here is really a two-dimensional problem, Ocola, Bauman, and Laane also calculated the two-dimensional potential energy surface for cyclopentane [31]. This is shown in Figure 18 and can be viewed either in terms of the bending and twisting coordinate or in terms of the radial and pseudorotational modes. Complementary density function and ab initio calculations have shown that the pseudoration is free or nearly free, with energy differences between the ten bent and ten twisted conformations being no greater than about 5 cm^−1^ or 0.14 kcal/mole. This was consistent with our earlier study for the effect of a ten-fold barrier to pseudorotation [32].

Laane et al. have also investigated the pseudorotation spectra of silacyclopentane [33] and 1,3-oxathiolane [34]. Both of these molecules hinder pseudorotation, since the bent structures are of higher energy than the twisted ones. The surface for 1,3-oxathiolane is also shown in Figure 18. A summary of early pseudorotational studies was published in 1972 [35]. In 1990, Rosas, Cooper, and Laane showed that molecular mechanics calculation did a fairly good job in predicting pseudorotational barriers [36].

### 2.7. Triplet-State Ring Puckering

**Figure 19:**
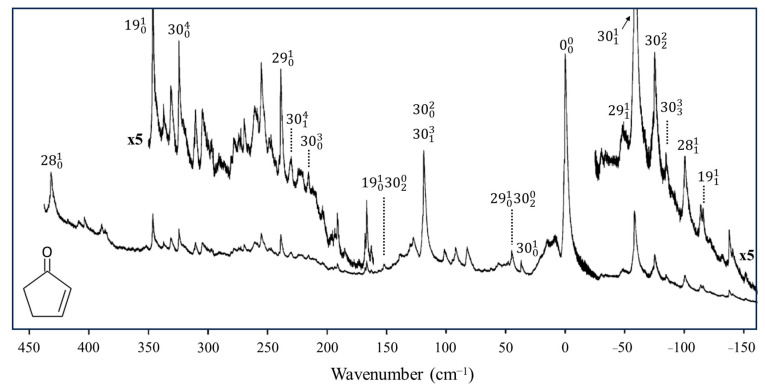
Cavity ringdown spectrum of 2-cyclopentenone.

**Figure 20:**
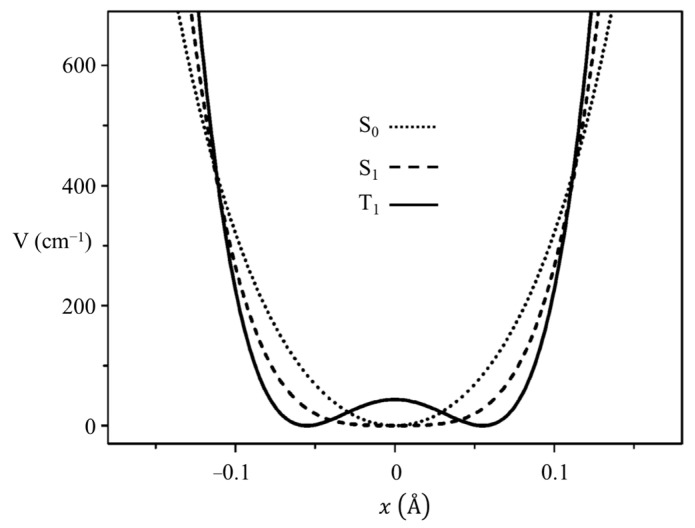
Ring-puckering potential energy functions for 2-cyclopentenone in three different electronic states.

In collaboration with the Drucker group, we investigated what the ring-puckering potential energy function looks like for the triplet state of 2-cyclopentene-1-one [37,38]. Figure 19 shows the cavity ringdown spectrum of this molecule. Vibration *v*_30_ is the puckering, with its lowest frequency for the triplet state at 37 cm^−1^. Fitting the data results in the one-dimensional function in Figure 20, where the potential functions derived for the ground state and singlet excited S_1_(n, π*) state are also shown. It is remarkable that the unpaired electrons in the triplet state produce a small barrier and result in a non-planar molecule.

### 2.8. Cyclohexane

**Figure 21:**
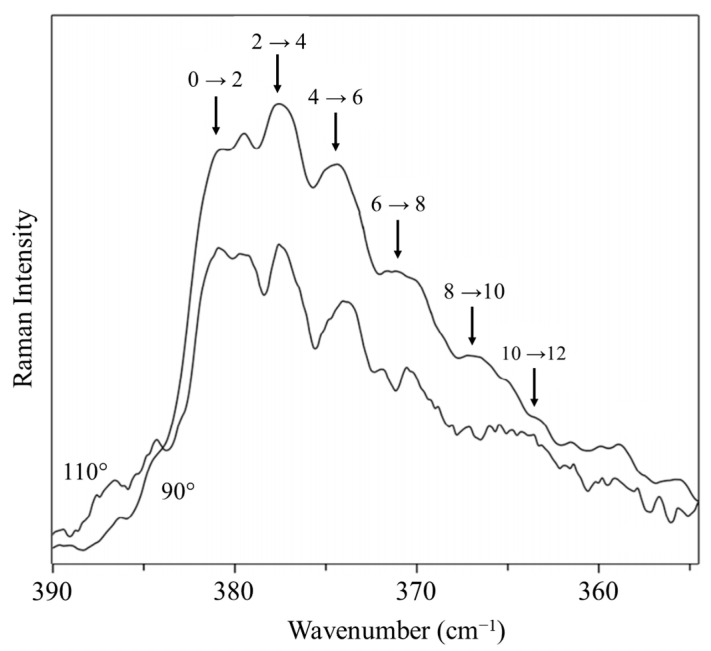
Vapor-phase Raman spectrum of heated cyclohexane vapor.

**Figure 22:**
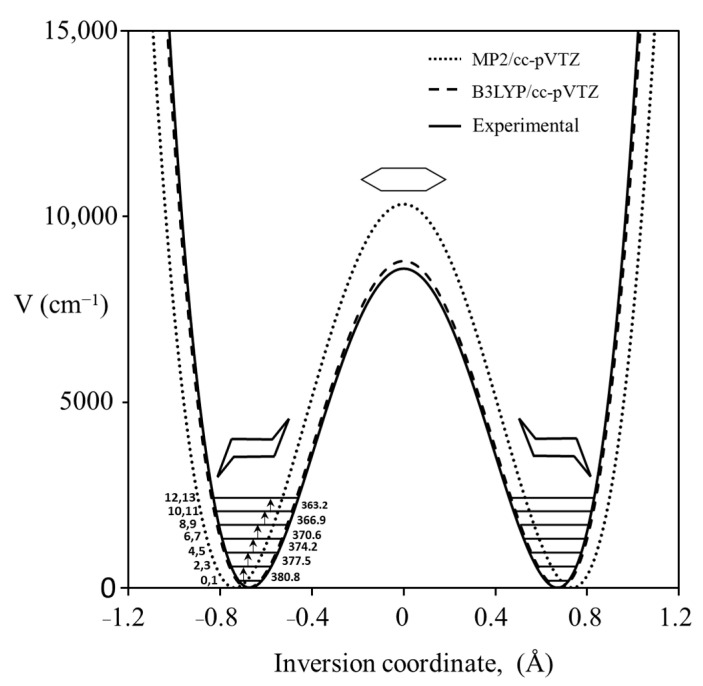
Cyclohexane potential energy function for the ring inversion.

We recorded the Raman spectrum for the bending vibration of cyclohexane [39], whose conformation has been investigated by many dozens of researchers. Figure 21 shows the spectrum, and Figure 22 shows the potential function based on Equation (2) calculated from the observed data for the out-of-plane vibration. The barrier to planarity for the calculated function is 8600 cm^−1^, and this agrees well with the DFT B3LYP/cc-pVTZ computed value of 8804 cm^−1^.

### 2.9. Internal Rotation

Hundreds, if not thousands, of studies have been carried out on internal rotations. For a single internal rotation angle, Θ, the infrared and Raman spectra resulting from these can generally be reproduced using a function of the form

(5) $$V=\sum V_{n}(1-cos(n\Theta )$$

The energy levels for simple three-fold or two-fold rotors were initially calculated using Mathieu tables. In 1972, Laane [40] developed the FORTRAN computer program VNCOSPX for calculating the energy levels for more complicated systems and fitting the experimental data. This program has been shared with dozens of labs around the world, and many more have written their own programs based on the matrix elements presented in our work. In 1977, a computer program was developed for even more complicated functions that also contained sine terms [41].

**Figure 23:**
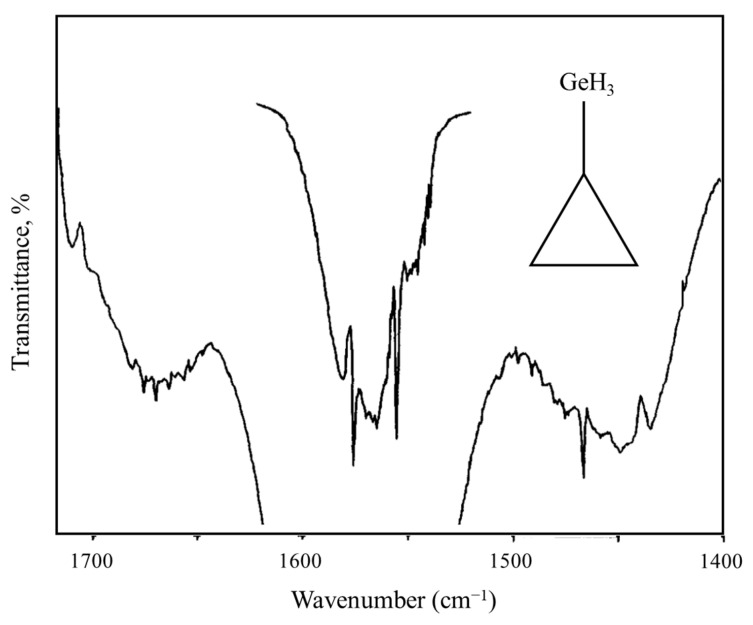
Internal rotation combination bands for cyclopropylgermane.

**Figure 24:**
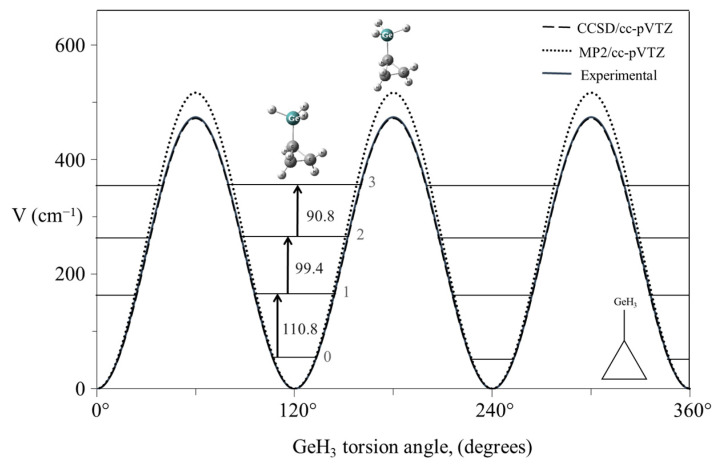
Experimental and theoretical potential energy functions for the internal rotation of cyclopropylgermane.

As an example of a system for a three-fold rotor, Figure 23 shows the mid-infrared combination band spectrum of cyclopropylgermane [42], and Figure 24 shows the experimental internal rotation PEF compared to those derived from theoretical calculations [43]. The torsional barrier can be seen to be about 450 cm^−1^.

**Figure 25:**
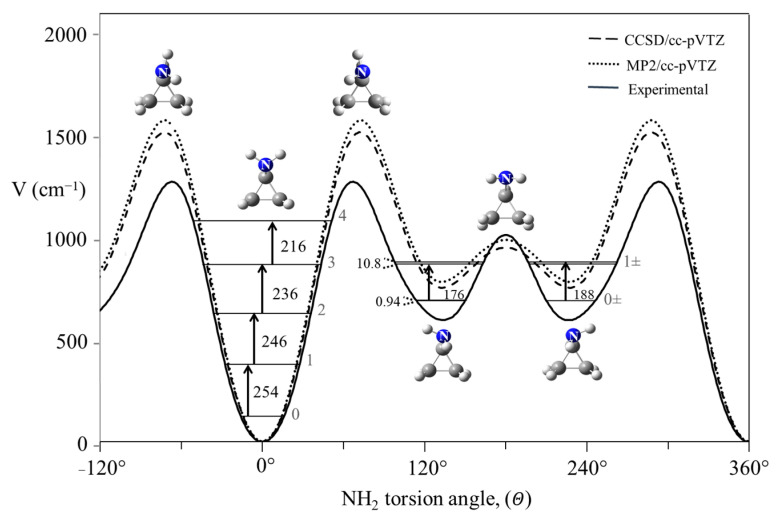
Internal rotation potential energy function for cyclopropylamine.

The potential energy function for cyclopropylamine shown in Figure 25 is quite different in that it has energy minima corresponding to two different conformations [43,44].

**Figure 26:**
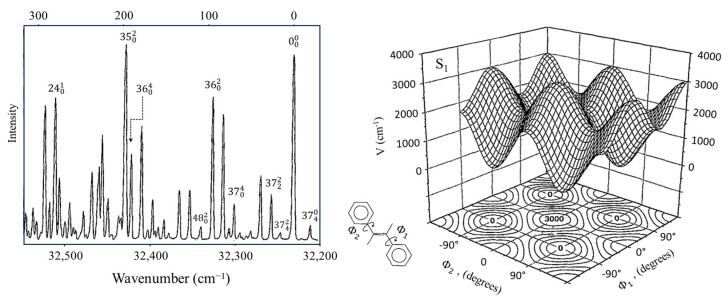
Fluorescence spectrum and two-dimensional internal rotation PES for *trans*-stilbene.

A more complex internal rotor case is that for the two internal rotations of *trans*-stilbene in its S_1_(π,π*) electronic excited state [45]. Future Nobel laureate Ahmed Zewail visited Laane’s research group in the 1990s at Texas A&M University, and after seeing the work on potential energy functions, he encouraged the study of this molecule, since his work at Caltech had raised some unanswered questions. Figure 26 shows the fluorescence excitation spectrum for this molecule and the two-dimensional internal rotation potential energy surface of Equation (6), which fits the data very well.

(6) $$V_{\left({Ф}_{1},{Ф}_{2}\right)}=\frac{1}{2}V_{2}\left(2+cos2{Ф}_{1}+cos2{Ф}_{2}\right)+V_{12}cos2{Ф}_{1}cos2{Ф}_{2}+\;{V\prime }_{12}sin2{Ф}_{1}sin2{Ф}_{2}$$

The torsional modes *v*_37_ and *v*_48_ have their lowest vibrational levels at 9 and 118 cm^−1^, respectively, for the excited state. This study helped clarify a long-standing uncertainty over the assignment of the electronic excitation spectra.

**Figure 27:**
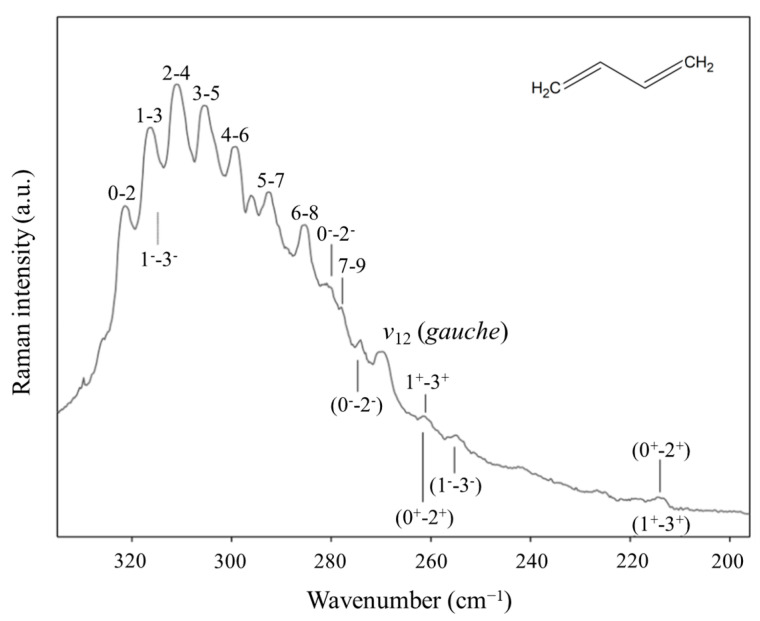
Low-frequency heated Raman spectrum of 1,3-butadiene.

**Figure 28:**
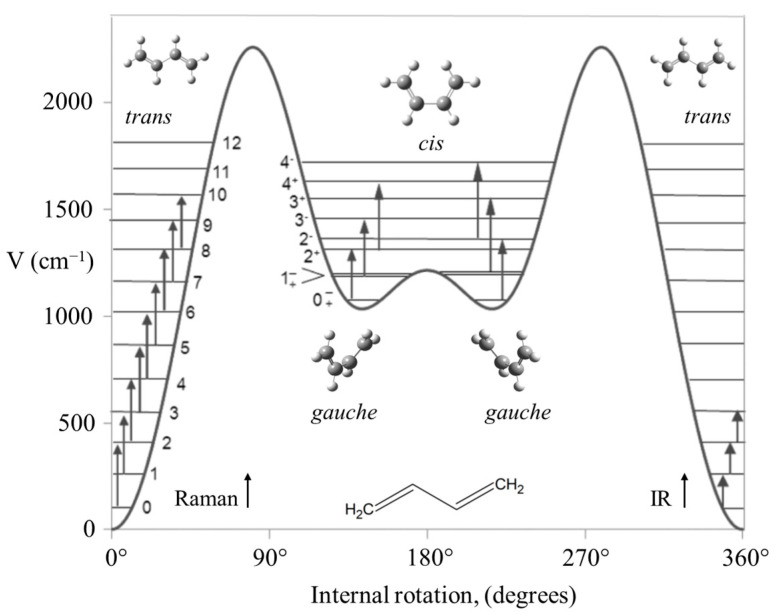
Potential energy function for the internal rotation of 1,3-butadiene.

Another long-standing problem had been the internal rotation of 1,3-butadiene before we investigated the high-temperature Raman spectrum of this molecule and its isotopic species [46,47]. The spectrum of the normal species is shown in Figure 27. The data allowed the determination of its internal rotation PEF in Figure 28, and this showed that the long-sought second isomer of this molecule is not a *cis* structure but a *gauche* conformer corresponding to two shallow minima. There exists a small barrier at the *cis* conformation. This experimental result agrees very well with theoretical computations.

### 2.10. π-Type Hydrogen Bonding

**Figure 29:**
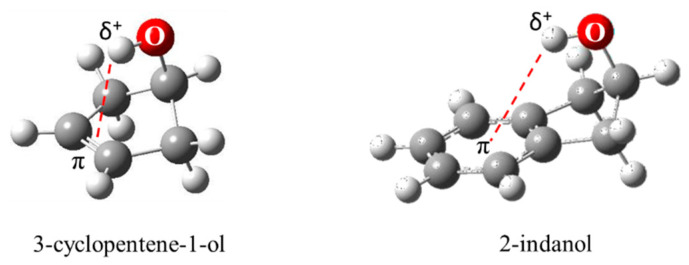
π-type hydrogen bonding of 3-cyclopenten-1-ol and 2-indanol.

**Figure 30:**
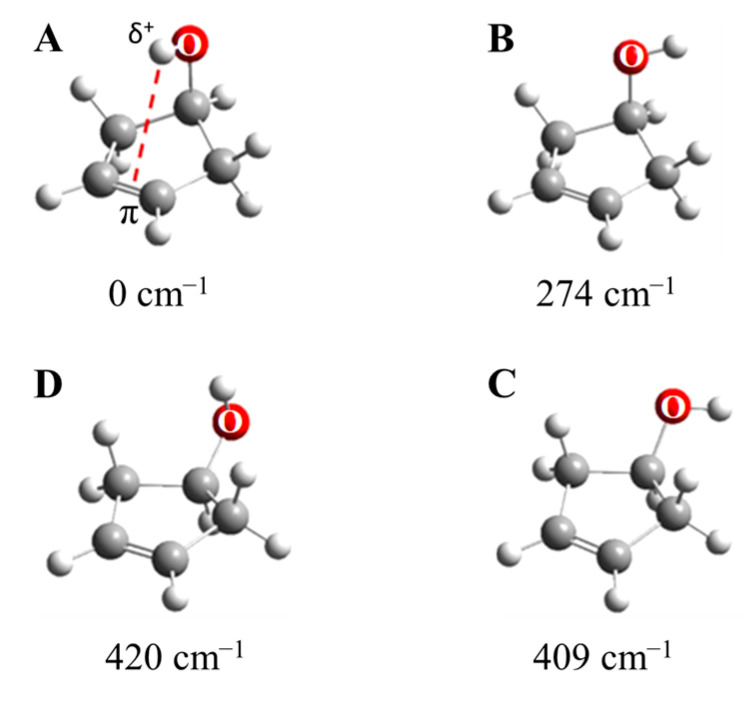
Conformers **A**, **B**, **C** and **D** of 3-cyclopenten-1-ol. The energies from CCSD/6-311++G(d,p) computations are shown.

**Figure 31:**
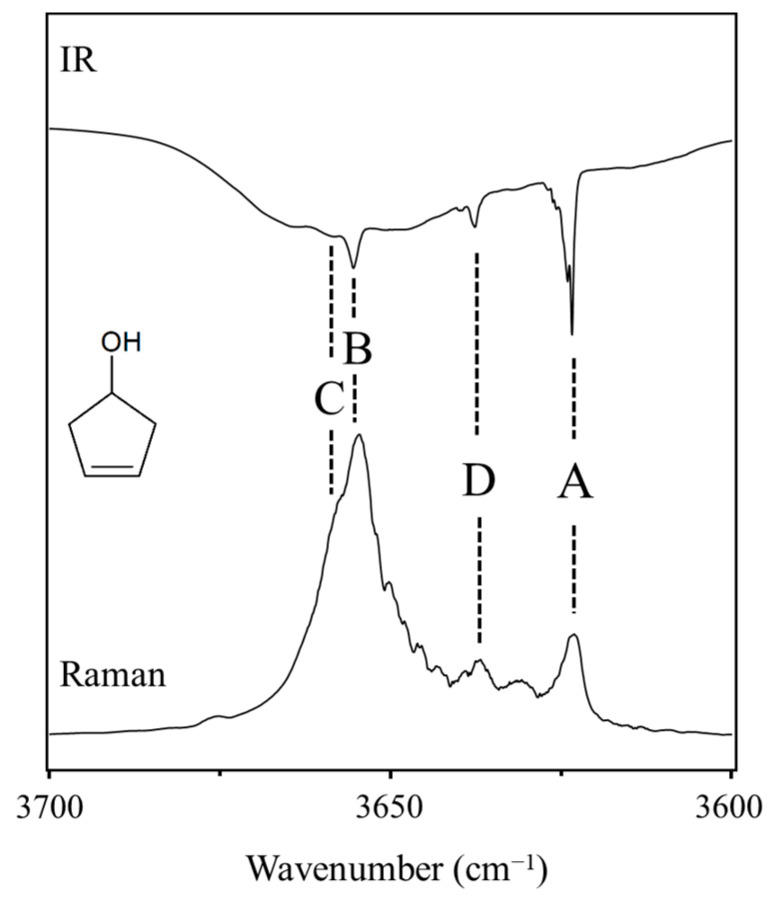
Infrared and Raman spectra of 3-cyclopenten-1-ol in the O-H stretching region showing bands from the four conformers **A**, **B**, **C** and **D**.

**Figure 32:**
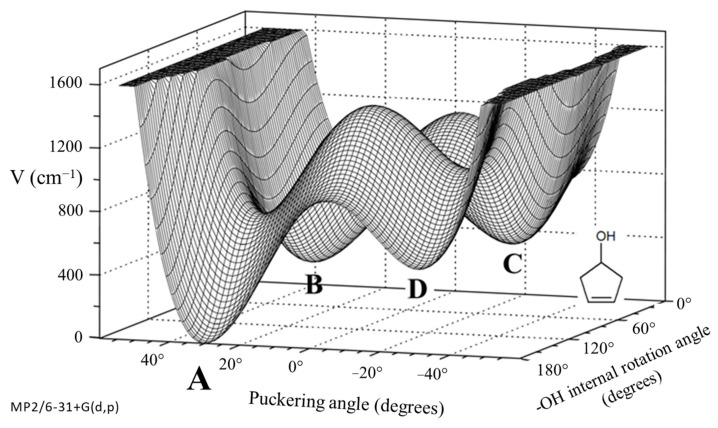
Two-dimensional potential energy surface for 3-cyclopenten-1-ol from ab initio MP2/6-31+G(d,p) computations. The minima correspond to the four conformations **A**, **B**, **C** and **D**.

As shown in Figure 29, 3-cyclopenten-1-ol and 2-indanol are capable of π-type hydrogen bonding. Figure 30 shows four possible conformations of 3-cyclopenten-1-ol along with their relative energies derived from ab initio CCSD/6-311++G(d,p) computations [48,49]. As can be seen, conformation **A** has the lowest energy, and this is due to π-type hydrogen bonding between the hydrogen atom of the OH group and the C=C double bond. This can only be achieved when both the ring-puckering coordinate and the internal rotation coordinate for the OH group allow the hydrogen atom to be close enough to the double bond to allow the π bonding. Figure 31 shows the infrared and Raman spectra in the O-H stretching region. Bands from all conformers can be seen, and that from the one with the π bonding is at the lowest frequency, as expected. Figure 32 shows the two-dimensional potential energy surface calculated for this molecule from ab initio MP2/6-31+G(d,p) computations. The minima occur where the puckering and internal rotation coordinates allow the π bonding.

**Figure 33:**
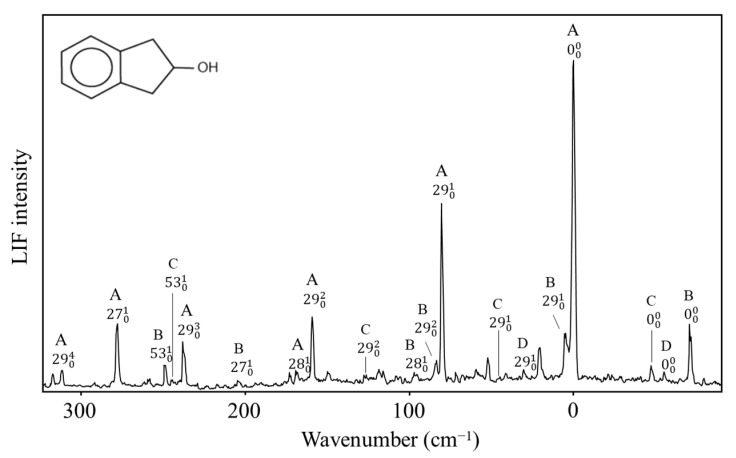
Fluorescence excitation spectrum of 2-indanol showing bands from its four conformers **A**, **B**, **C** and **D**.

The two-dimensional surface calculated for 2-indanol is similar [50]. Al-Saadi, Wagner, and Laane have recorded the fluorescence excitation spectrum for this molecule, as shown in Figure 33. The spectra of all four conformers, **A**, **B**, **C**, and **D**, can be seen. Their relative energies are similar to those of the 3-cyclopenten-1-ol.

We have also investigated the spectra of several other molecules with the π-type hydrogen bonding [51,52,53], which in each case lowers their conformational energy.

### 2.11. Pyridine and Sir Harry Kroto

**Figure 34:**
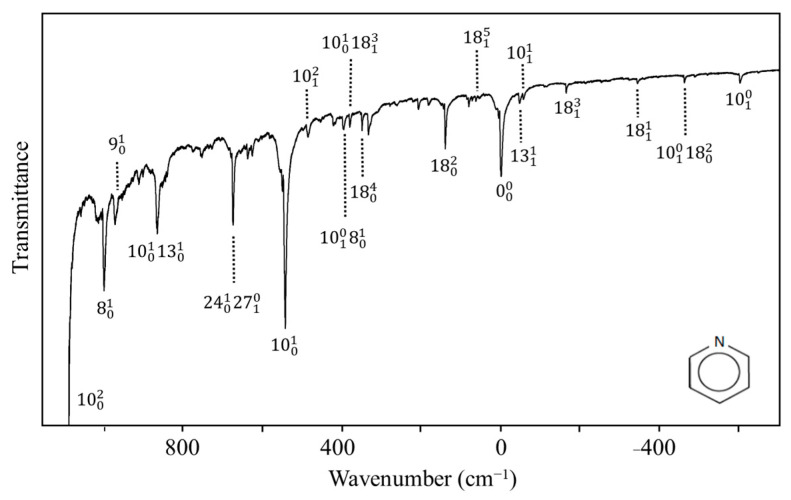
Ultraviolet absorption spectrum of pyridine.

**Figure 35:**
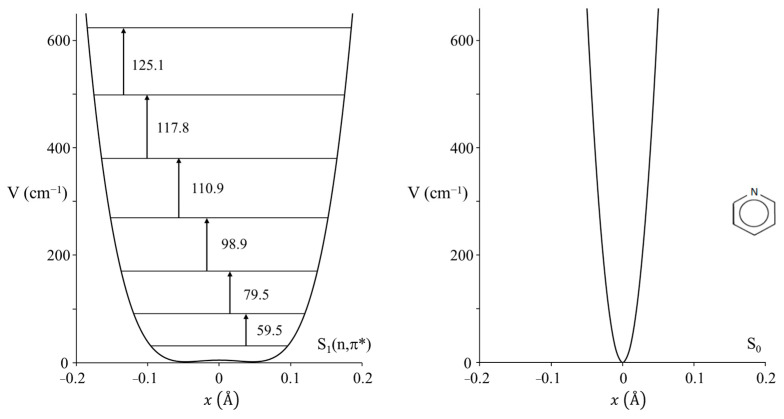
Ring-bending potential energy function for pyridine in its S_1_(n,π*) state (**left**) and compared to that in the ground state (**right**).

Nobel laureate Sir Harry Kroto visited us in the early 2000s and told us about his work on pyridine [54] when he was a post-doc in Ottawa in the 1970s. He had recorded the ultraviolet absorption spectrum of this molecule but had not fully assigned the spectrum. Since we had a high-resolution Bomem DA8.02 FT spectrometer available, this molecule along with its d_5_ isotopic species was reinvestigated by our research group [55]. The spectrum for the d_0_ molecule is shown in Figure 34. The PEF derived for the out-of-plane ring-bending vibration in its S_1_(n, π*) state is shown in Figure 35, which also compares it to the function for the ground state. As can be seen, the electronic transition to the antibonding π orbital greatly reduces the rigidity of the pyridine ring. In fact, there is a tiny 4 cm^−1^ barrier to planarity for the excited state.

Laane’s research group has also investigated the ultraviolet absorption spectra of several fluoropyridines [56,57]. Some have small barriers to planarity for the excited states, while others are planar but are still not very rigid.

## 3. Conclusions

Compared to the potential energy functions described in this paper, the harmonic oscillator is indeed boring. We have demonstrated in the present paper how one- and two-dimensional potential energy functions for selected systems can be determined very accurately to fit experimental data and therefore lead to detailed information on molecular structures and energetics. As an example, in our Table 2 we show the remarkably good fit between experimental far-infrared frequencies and the values calculated from the simple potential function of Equation (2). Similarly excellent fits were achieved for the other potential functions discussed in the present work, and these can be seen in the references provided. Physical chemistry textbooks generally show solutions of the wave equation for the harmonic oscillator, but these are not terribly convincing since only one parameter (k in $kx^{2}$) is used to fit a single vibrational frequency. In our studies described in this paper, the two-parameter potential energy function in Equation (2) typically fits more than a dozen observed frequencies extremely well.

## Figures and Tables

**Table 1:** Inversion barriers for carbonyl groups in electronic excited states.

	C=O Inversion Barrier (cm^−1^)
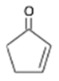 2-cyclopenten-1-one	0
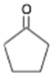 Cyclopentanone	672
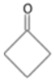 Cyclobutanone	939
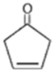 Cyclopentene-1-one	2149

**Table 2:** Observed and calculated ring-puckering frequencies for 2,3-dihydrofuran.

	Frequency (cm^−1^)			Relative Intensity	
Transition	Observed	Calculated	∆	Observed	Calculated
*v*_T_ = 0					
1–2	88.8	88.6	0.2	0.80	0.90
2–3	83.5	83.5	0.0	0.76	0.87
3–4	103.8	103.6	0.2	(1.00)	(1.00)
4–5	114.9	114.8	0.1	0.77	0.83
5–6	124.9	124.8	0.1	0.75	0.61
6–7	133.3	133.4	−0.1	0.50	0.41
7–8	141.1	140.9	0.2	0.40	0.26
8–9	147.4	147.7	−0.3	0.22	0.15
9–10	152.7	153.9	−1.2	0.17	0.09
10–11	160.4	159.9	0.5	0.06	0.05
0–3	191.2	191.1	0.1	0.15	0.27
1–4	275.9	275.6	0.3	0.20	0.11
2–5	301.8	301.8	0.0	0.06	0.08
3–6	343.2	343.2	0.0	0.06	0.05
4–7	372.2	372.9	−0.7	0.05	0.03
5–8	398.9	398.1	0.8	0.04	0.02
6–9	422.1	422.0	0.1	0.02	0.01

V (cm^−1^) = $1.019\times 10^{6}x^{4}-1.946\times 10^{4}x^{2}$.

**Table 3:** Comparison of experimental barriers and $x_{min}$ and $\theta_{min}$ values with those from CCSD/ccpVTZ and MP2/cc-pVTZ computations for cyclobutane and related molecules.

	Experimental Fit				Theoretical				
	$V(x^{6},x^{4},x^{2})$ ^a^			CCSD/cc-pVTZ			MP2/cc-pVTZ		
Molecule	Barrier	$x_{min}$	$\theta_{min}$	Barrier	$x_{min}$	$\theta_{min}$	Barrier	$x_{min}$	$\theta_{min}$
	cm^−1^	Å	Degrees	cm^−1^	Å	Degrees	cm^−1^	Å	Degrees
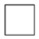 C_4_H_8_	510	±0.142	±29.6°	586	±0.142	±29.5°	821	±0.153	31.9°
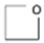 C_4_H_7_O	15	±0.062	±13.4°	0	±0.001	±0.2°	21	±0.069	14.8°
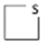 C_4_H_7_S	273	±0.141	±26.9	243	±0.133	±25.5°	412	±0.152	29.0°
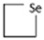 C_4_H_7_Se	378	±0.162	±30.4°	339	±0.148	±27.8°	512	±0.164	30.9°
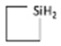 C_4_H_7_SiH_2_	440	±0.163	±31.9°	472	±0.163	±31.7°	654	±0.176	34.5°
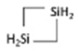 C_4_H_7_(SiH_2_)_2_	87	±0.130	±22.3°	89	±0.130	±22.4°	160	±0.150	25.8°
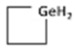 C_4_H_7_GeH_2_	---	---	---	409	±0.156	±30.1°	567	±0.169	32.6°
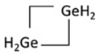 C_4_H_7_(GeH_2_)_2_	---	---	---	1	±0.047	±7.8°	24	±0.104	17.1°
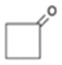 C_4_H_4_O	2	±0.043	±4.6°	36	±0.073	±15.5°	114	±0.097	20.8°

^a^ Using $V=ax^{4}+bx^{2}+cx^{6}.$

**Table 4:** Relative magnitudes of the negative parameter b in Equation (2) reflecting the torsional contribution from the anomeric effect.

Molecule	Linkage	Relative b Value	$V_{anomeric}$ ^*a*^ (kcal/mole)
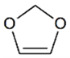	OCO	1.00	5.97 ^*b*^
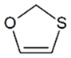	OCS	0.82	4.90
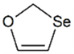	OCSe	0.66	3.94
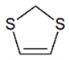	SCS	0.60	3.58
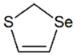	SCSe	0.45	2.69
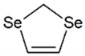	SeCSe	0.32	1.91

*^a^* Estimated torsional potential energy from the anomeric effect. *^b^* Magnitude from reference 22 based on MM3 molecular mechanics calculations.

## Data Availability

The data are available in this article.
